# Ring of Change: CDC48/p97 Drives Protein Dynamics at Chromatin

**DOI:** 10.3389/fgene.2016.00073

**Published:** 2016-05-03

**Authors:** André Franz, Leena Ackermann, Thorsten Hoppe

**Affiliations:** Cologne Excellence Cluster on Cellular Stress Responses in Aging-Associated Diseases, Institute for Genetics, University of CologneCologne, Germany

**Keywords:** CDC48, p97, ubiquitin, SUMO, chromatin, replication, DNA repair

## Abstract

The dynamic composition of proteins associated with nuclear DNA is a fundamental property of chromosome biology. In the chromatin compartment dedicated protein complexes govern the accurate synthesis and repair of the genomic information and define the state of DNA compaction in vital cellular processes such as chromosome segregation or transcription. Unscheduled or faulty association of protein complexes with DNA has detrimental consequences on genome integrity. Consequently, the association of protein complexes with DNA is remarkably dynamic and can respond rapidly to cellular signaling events, which requires tight spatiotemporal control. In this context, the ring-like AAA+ ATPase CDC48/p97 emerges as a key regulator of protein complexes that are marked with ubiquitin or SUMO. Mechanistically, CDC48/p97 functions as a segregase facilitating the extraction of substrate proteins from the chromatin. As such, CDC48/p97 drives molecular reactions either by directed disassembly or rearrangement of chromatin-bound protein complexes. The importance of this mechanism is reflected by human pathologies linked to p97 mutations, including neurodegenerative disorders, oncogenesis, and premature aging. This review focuses on the recent insights into molecular mechanisms that determine CDC48/p97 function in the chromatin environment, which is particularly relevant for cancer and aging research.

## Introduction

DNA is the most precious resource of an organism. Its faithful transmission to following generations is of major importance for an individual. Elaborate surveillance mechanisms are required to guard the genome, since large amounts of heterogeneous protein complexes are active at the DNA. Thus, DNA is packaged into highly dynamic chromatin structures for efficient space usage. This involves different histone variants as well as complex protein cohorts that allow for genome function (Misteli, 2007; Cutter and Hayes, 2015). Dependent on cell type, cell cycle phase, environmental cues, or aging status, multisubunit replication and transcription machineries access chromatin and thereby challenge chromosome integrity. In addition, various maintenance and repair mechanisms are active that keep chromatin intact. To ensure genome stability these processes need to be coordinated and tightly controlled in time and space. Within complex protein agglomerations specific proteins have to be recruited or removed to allow a given process to continue. The underlying molecular signaling is predominantly triggered by post-translational modifications (PTMs) of target proteins.

**FIGURE 1:**
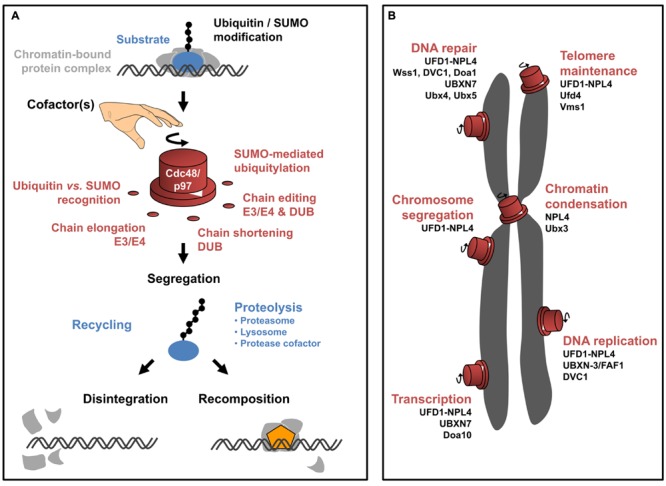
**CDC48/p97 function in chromatin-associated processes. (A)** Schematic illustration of molecular mechanisms underlying CDC48/p97 activity. CDC48/p97 (red) recognizes chromatin-bound substrates (blue) that are conjugated to ubiquitin or SUMO (black circles). The modification with ubiquitin and SUMO can come in different flavors (exemplified by a chain of molecules). Both molecules can be conjugated as a monomeric moiety or a chain of several molecules. The linkage in between molecules of a ubiquitin chain is variable depending on the internal lysine-(K)-residue used for chain extension (indicated by the angle between ubiquitin molecules of a chain). As such, diverse linkages are capable of defining distinct signaling events (referred to as ‘ubiquitin-code’). Moreover, SUMO-dependent ubiquitylation gives rise to hybrid chains. Depending on the exact modification of the substrates, diverse cofactors facilitate substrate recognition and/or processing of the ubiquitin/SUMO modification by extending, removing, or internal remodeling of the chain. This is probably important to define the directionality of the CDC48/p97 reaction. Eventually, CDC48/p97 segregase activity is required to mobilize the substrate from higher order protein complexes (light gray). On one hand the substrate can be recycled, probably involving hydrolysis of the modification. Otherwise, the substrate can be terminally degraded involving the proteasome, lysosome, or proteolytic cofactors. Extraction of the substrate can promote two distinct outcomes. Disintegration of the protein complex can result in its inactivation (bottom left). Alternatively, extraction of the substrate can disclose the binding site of another factor (orange) thus facilitating the directed progression of the reaction (bottom right). **(B)** Schematic overview of CDC48/p97-dependent pathways in the context of eukaryotic chromosomes (gray). CDC48/p97 (red) possesses molecular switch properties, driving molecular reactions in distinct chromatin-associated processes. The involvement of respective CDC48/p97 cofactors is listed below the indicated pathways.

The ATPase CDC48/p97 (also known as VCP in human) is a central factor that integrates recognition, modification and execution of molecular processes mediated by ubiquitin (Ghislain et al., 1996; Meyer et al., 2000; Dai and Li, 2001; Wojcik et al., 2004) or ubiquitin-like molecules (Krick et al., 2010; Bandau et al., 2012; den Besten et al., 2012; Nie et al., 2012; Bergink et al., 2013; Køhler et al., 2013, 2015). CDC48/p97 forms homo-hexameric ring-like particles, which undergo extensive conformational changes upon ATP-hydrolysis (Rouiller et al., 2002; Banerjee et al., 2016). These intramolecular changes drive the mechanistic function of CDC48/p97, which is best described as segregase activity (Rape et al., 2001; Braun et al., 2002; Shcherbik and Haines, 2007). While the precise molecular mechanism of substrate handling is controversial (Stolz et al., 2011; Barthelme and Sauer, 2015), cumulating evidence suggests that the ATP-dependent conformational rearrangements account for partial unfolding of substrates (Beskow et al., 2009; Godderz et al., 2015; Song et al., 2015), thereby promoting their segregation from multimeric protein assemblies. Following the recognition of target proteins that are marked by ubiquitin, SUMO or both, CDC48/p97 mobilizes the modified substrates from higher order protein complexes, resulting in their inactivation by breaking off the molecular context and/or promoting subsequent proteolytic turnover (**Figure 1**). The cellular processes that rely on CDC48/p97 segregase activity are diverse (Franz et al., 2014), ranging from degradation of damaged proteins associated with the endoplasmic reticulum (ER, ERAD; Ye et al., 2001; Braun et al., 2002; Jarosch et al., 2002; Rabinovich et al., 2002) or mitochondria (MAD; Heo et al., 2010; Hemion et al., 2014; Fang et al., 2015), ribosome-associated quality control (Ossareh-Nazari et al., 2010; Brandman et al., 2012; Verma et al., 2013) to lipid droplet metabolism (Olzmann et al., 2013), and lysosomal proteolysis (Ren et al., 2008; Ju et al., 2009; Krick et al., 2010; Tresse et al., 2010; Ritz et al., 2011; Buchan et al., 2013). Recently, most attention has been paid to the role of CDC48/p97 in the directed modulation of chromatin-associated protein complexes (Vaz et al., 2013; Dantuma et al., 2014). Herein, fundamental cellular processes such as DNA synthesis and DNA repair as well as transcriptional regulation require CDC48/p97.

Given the growing number of cellular pathways relying on CDC48/p97 function, it appears obvious that independent regulatory mechanisms are required to control the diverse molecular activities. In this context, cofactors provide specificity toward defined CDC48/p97 pathways (Decottignies et al., 2004; Hartmann-Petersen et al., 2004; Medicherla et al., 2004; Schuberth et al., 2004; Wang et al., 2004; Neuber et al., 2005; Park et al., 2005; Richly et al., 2005; Schuberth and Buchberger, 2005; Song et al., 2005; Ritz et al., 2011). Most cofactors interact with CDC48/p97 via conserved binding motifs and provide additional molecular properties that assist in substrate recognition, processing, or regulation of ATPase activity. Substrate recruiting cofactors harbor dedicated domains that recognize conjugated ubiquitin or SUMO, thereby facilitating substrate binding (Kloppsteck et al., 2012; Meyer and Weihl, 2014; Buchberger et al., 2015). Processing cofactors alter the length or topology of ubiquitin or SUMO marks, either by extending (E3–E4 enzymes), shortening (ubiquitin/SUMO hydrolases), or remodeling (also called editing, combined E3–E4 and hydrolase activities) the conjugates (Koegl et al., 1999; Hoppe, 2005; Rumpf and Jentsch, 2006; Jentsch and Rumpf, 2007; Kuhlbrodt et al., 2011; Heride et al., 2014). Other cofactors regulate CDC48/p97 ATPase activity (Trusch et al., 2015; Zhang et al., 2015) thus controlling substrate processing. Cofactors themselves can provide another layer of associated factors (termed accessory factors), thereby defining CDC48/p97 function (Alexandru et al., 2008; Sowa et al., 2009; Balakirev et al., 2015; Raman et al., 2015; **Figure 1**; **Table 1**).

**Table 1:** CDC48/p97 substrates in the chromatin environment.

Cdc48/p97-dependent process	Substrate(s)	Cofactor/Accessory factors	Experimental system	Reference
**DNA replication**				
Replication fork progression, G2/M checkpoint	n.d.	DVC1	Mammalian cells, patient cells	Lessel et al., 2014
Replication licensing	CDT-1, CDC-45-GINS	UFD-1, NPL-4, UBXN-3/FAF1	*C. elegans, X. laevis*, mammalian cells	Mouysset et al., 2008; Franz et al., 2011, 2016
Replication stress	FANCI, FANCD2	DVC1	Mammalian cells	Gibbs-Seymour et al., 2015
Replication stress, DNA damage tolerance (DDT)	Polη, a.o.	DVC1	Mammalian cells, *C. elegans*	Davis et al., 2012; Mosbech et al., 2012
Replication termination	MCM7	Dia2	*S. cerevisiae, X. laevis*	Maric et al., 2014; Moreno et al., 2014; Maculins et al., 2015
**DNA repair**				
Diverse genotoxic insults	SUMO/Ubiquitin-conjugates	Ufd1, Npl4	*S. cerevisiae*	Nie et al., 2012
Diverse genotoxic insults	SUMO/Ubiquitin-conjugates	Ufd1, Npl4, Rfp1, Pli1	*S. pombe*	Køhler et al., 2013
DNA damage response (DDR)	Top1, SUMO-conjugates	Wss1, Doa1	*S. cerevisiae*	Balakirev et al., 2015
DNA-double strand break repair	L3MBTL1	UFD1, NPL4	Mammalian cells, *C. elegans*	Acs et al., 2011
DNA-double strand break repair	SUMO-Rad52	Ufd1	*S. cerevisiae*, mammalian cells	Bergink et al., 2013
DNA-double strand break repair	Ubiquitin-(K48)-conjugates	UFD1, NPL4	Mammalian cells	Meerang et al., 2011
DNA-double strand break repair	DNA-PKcs	n.d.	Mammalian cells	Jiang et al., 2013
DNA-protein crosslink (DPC)	Top1, a.o.	Wss1	*S. cerevisiae*	Stingele et al., 2014
PCNA-dependent response to UV-light	n.d.	DVC1, mono-ubiquitylated PCNA	Mammalian cells	Centore et al., 2012
UV-light induced protein turnover	CSB	UFD1, UBXN7, CUL4	Mammalian cells	He et al., 2016
UV-light induced protein turnover	CDT1, SET8	UFD1, NPL4, a.o.	Mammalian cells, *X. leavis*	Raman et al., 2011
UV-light induced protein turnover	DDB2, XPC	UFD1, NPL4, UBXN7, CUL4	Mammalian cells	Puumalainen et al., 2014
UV-light induced protein turnover	Rbp1	Ufd1, Npl4, Ubx4, Ubx5, Cul3	*S. cerevisiae*	Verma et al., 2011
**Transcription**				
Histone ubiquitylation	Histone 2B	Ubx3	*S. cerevisiae*, mammalian cells	Bonizec et al., 2014
Mating-type switch	α2	Ufd1, Npl4, Doa10, a.o.	*S. cerevisiae*	Wilcox and Laney, 2009
Transcriptional inactivation	HIF1α	UBXN7, CUL2, VHL	Mammalian cells	Alexandru et al., 2008
Transcriptional regulation	LexA-VP16, Met4, R-Smads	Ufd1, Npl4	*S. cerevisiae*	Ndoja et al., 2014
Heterochromatin decondensation	CenH3	Ufd1, Npl4	*A. thaliana*	Merai et al., 2014
**Telomere maintenance**				
Telomerase efficiency	Cdc13	Vms1	*S. cerevisiae*	Baek et al., 2012
Telomerase efficiency	Est1	Ufd1, Npl4, Ufd4	*S. cerevisiae*	Lin et al., 2015
**Sister-chromatid segregation**				
Anaphase degradation	n.d.	Ubx4	*S. cerevisiae*	Chien and Chen, 2013
Chromatin decondensation/congression	Aurora-B	Ufd1, Npl4	*X. leavis*, mammalian cells	Ramadan et al., 2007; Dobrynin et al., 2011
Meiosis	AIR-2	n.d.	*C. elegans*	Sasagawa et al., 2012
**Others**				
Global analysis	SUMO-conjugates	Ufd1, STUbL	*S. pombe*	Køhler et al., 2015

*The table lists the identified chromatin-associated substrates of CDC48/p97, sorted by their functional relevance in indicated cellular processes. In addition, the involvement of cofactors and/or accessory factors is displayed along with the experimental system that was used in the respective publication (a.o. and others, n.d. not determined).*

The requirement of an organism for CDC48/p97 originates from the variety of processes that depend on its segregase activity. Hence, alterations in CDC48/p97 protein expression or mutations are associated with different diseases including neurodegeneration or premature aging (Partridge et al., 2003; Johnson et al., 2010; Nalbandian et al., 2011; Franz et al., 2014). Moreover, CDC48/p97 overexpression is associated with different cancer types connected with poor prognosis (Fessart et al., 2013). This is intelligible given the diverse chromatin related pathways, like replication or DNA repair that CDC48/p97 is associated with. Since each of these pathways is highly related to tumor formation, p97 constitutes a reasonable target for anticancer therapy (Balch et al., 2008) and first inhibitors are already tested in clinical trials (Deshaies, 2014; Chapman et al., 2015). This review provides an overview on the fundamental role of CDC48/p97 in controlling activity and dynamics of protein complexes at the chromatin. For simplicity, we will refer to spelling of conserved human orthologs throughout the article, unless otherwise stated.

## DNA Replication is Driven By Dynamic Composition of Protein Complexes

The faithful duplication of genomic information during S phase of the cell cycle is a complex biological process involving the highly ordered cascade of numerous replication factors at the chromatin (Masai et al., 2010; Fragkos et al., 2015). DNA synthesis is initiated at origins of replication, which serve as assembly platforms for DNA synthesis factories, termed replisomes. Herein, the concerted activity of origin recognition complex (ORC), CDC6, and CDT1 is required to load the replicative DNA helicase, the Mini-chromosome-maintenance (MCM) complex onto DNA. Together these factors constitute the pre-replicative complex (pre-RC). As pre-RCs do not perform helicase activity yet, pre-RC assembly is considered as licensing of DNA replication. Interestingly, inaccurately assembled pre-RCs can disassemble from DNA implicating that replication licensing involves quality control mechanisms and iterative loading events (Chen et al., 2007; Xouri et al., 2007; Frigola et al., 2013; Duzdevich et al., 2015). Subsequent to MCM assembly, the pre-RC components are dispensable and consequently inactivated. Origins actively synthesizing DNA are characterized by recruitment of further factors, including CDC45 and the go-ichi-ni-san (GINS) complex (Gambus et al., 2006; Moyer et al., 2006; Ilves et al., 2010). The presence of CDC45 and GINS thus characterizes active replisomes. During ongoing DNA synthesis and particularly close to completion of DNA replication converging replication factories collide and are considered to require regulated disassembly (Maric et al., 2014; Moreno et al., 2014). These processes exemplify that the composition of replication factories is highly dynamic throughout the regular replication program and, moreover, responsive to genotoxic insults that might threaten genome stability (Sirbu et al., 2013; Alabert et al., 2014; Dungrawala et al., 2015; Raschle et al., 2015). Intriguingly, CDC48/p97 has been shown to be essential for DNA replication in eukaryotes by regulating the abundance of several replication factors at distinct time points (**Figure 1**; **Table 1**).

### CDC48/p97-mediated Control of DNA Replication Licensing and Fork Progression

The functional relevance of CDC48/p97 in DNA synthesis was first shown in *Caenorhabditis elegans* (*C. elegans*). RNAi-mediated depletion of CDC48/p97 or the dimeric cofactor UFD-1-NPL-4 caused replication defects accompanied with collapsed forks and formation of DNA repair foci (Mouysset et al., 2008). This initial observation of compromised DNA synthesis upon inactivation of the CDC48/p97^UFD-1-NPL-4^ complex was further addressed in a follow-up study, identifying chromatin-associated CDT-1 is the primary substrate (Franz et al., 2011; Raman et al., 2011). Herein, the abundance of the licensing factor CDT-1 on chromatin relies on CDC48/p97 activity during initiation of DNA replication (Franz et al., 2011). In addition, CDT-1 stabilization on mitotic chromatin (correlating to G1 phase), coincides with chromatin-retention of CDC-45 and the GINS complex (Franz et al., 2011). A genetic interaction screen identified the UBX-domain protein UBXN-3 as a specialized cofactor enhancing substrate recognition by CDC48/p97 during DNA replication (Franz et al., 2016). Indeed, *in vivo* and *in vitro* protein interaction analysis confirmed that UBXN-3 provides substrate recognition toward CDT-1 and other ubiquitylated proteins (Franz et al., 2016). Analysis of individual replication forks in human cell lines revealed that siRNA-mediated depletion of the human ortholog FAF1 causes severely impaired replication fork progression associated with elevated frequency of replication fork stalling and firing of dormant origins. In fact, CDT1 protein appears to be the primary target of CDC48/p97^FAF1^ also in human cells, as indicated by genetic suppression of replication defects upon codepletion and *in vivo* binding studies (Franz et al., 2016). Taken together, CDC48/p97^UFD1-NPL4^, in complex with the substrate recognition module UBXN-3/FAF1, controls replication fork progression by restraining the abundance of CDT1 during replication licensing.

It should be noted that the regulatory mechanism depicted above is specific to the G1 phase of the cell cycle (Ballabeni et al., 2004; Franz et al., 2011). In contrast CDT1 protein levels are also under control during the ensuing S phase, involving PCNA and Cullin-based E3 ligases (Zhong et al., 2003; Arias and Walter, 2007; Havens and Walter, 2009; Sugimoto et al., 2009; Coleman et al., 2015). S phase degradation of CDT1 is considered as fundamental in preventing over-replication in one cell cycle as well as avoidance of chromosomal rearrangements (Davidson et al., 2006; Tatsumi et al., 2006). In response to DNA damage, CDT1 chromatin extraction and degradation also involves CDC48/p97 activity (Hu et al., 2004; Jin et al., 2006; Raman et al., 2011). In contrast to the licensing factors ORC and Cdc6, Cdt1 is required for break-induced replication in yeast (Lydeard et al., 2010). The exact requirement of CDT1 and its subsequent inactivation during DNA repair, however, remains elusive.

Interestingly, another thus far unappreciated cofactor of CDC48/p97 has attracted attention as a critical regulator in cellular pathways ensuring genome integrity (Stingele et al., 2015). Two studies could show that mutations in DVC1 [also called Spartan (SPRTN) or C1orf124 in humans, functionally related to Wss1 in *Saccharomyces cerevisiae*] are causative for genome instability phenotypes cumulating in hepatocellular carcinoma and progeria (Lessel et al., 2014; Maskey et al., 2014; **Figure 1**; **Table 1**). Patient cells expressing dysfunctional DVC1 show hallmarks of genomic instability, which is accompanied by aberrant replication fork velocity along with excessive replication stress. Furthermore, patient cell lines escape the cell cycle control by G2/M checkpoint, which usually halts the transition into mitosis until damage is repaired. Importantly, human cells exclusively expressing disease-related DVC1 mutations phenocopy the observations made in primary cells (Lessel et al., 2014). Identification of respective target substrates will decipher, which aspect of DNA replication is controlled by DVC1. Indeed DVC1 could be shown to colocalize with DNA replication factories in synchronized but otherwise untreated mammalian cells (Davis et al., 2012). Its functional relevance, however, became particularly important upon treatment with various types of genotoxic agents, which triggered the DVC1-dependent recruitment of CDC48/p97 to sites of DNA damage (Centore et al., 2012; Davis et al., 2012; Mosbech et al., 2012). Intriguing insights into the mechanistic function of DVC1’s cognate Wss1 in chromatin-associated protein degradation have recently been reported in the context of replication-coupled DNA repair (Stingele et al., 2014; Balakirev et al., 2015). Herein, Wss1 protease was identified to specifically mediate the processing of DNA-protein crosslinks (DPC) in a thus far overlooked repair pathway that presumably also underlies genome instability in DVC1 mutant cells (**Figure 1**; **Table 1** and references therein). Accordingly, disease-causing mutations locate in the domain encoding the predicted DVC1 protease (SprT) domain (Lessel et al., 2014); however, a chromatin-directed protease activity of DVC1 awaits affirmation. The mechanistic details of DPC-repair will be discussed in the respective paragraph on DNA-repair pathways.

### Termination of DNA Replication Requires CDC48/p97 Activity

Until recently, the molecular mechanisms underlying the termination of DNA replication in metazoans was only scarcely described (Dewar et al., 2015). Thus, the identification of CDC48/p97 in the release of the MCM helicase in complex with CDC45 and GINS (collectively termed CMG complex) at sites of replication termination was astonishing (Maric et al., 2014; Moreno et al., 2014). Yeast cells or *Xenopus* egg extracts that are depleted for CDC48/p97 are defective in the disassembly of the CMG complex at the end of the cell cycle when replication forks collide with high frequency. Both studies show that selective poly-(K48)-ubiquitylation of the MCM7 subunit is required to trigger CMG release (Maric et al., 2014; Moreno et al., 2014). Interfering with polyubiquitylation as well as CDC48/p97 activity, result in accumulation of DNA structures comparable to pharmacological inhibition of termination (Moreno et al., 2014). Moreover, ubiquitylation of MCM7 depends on active progression through S phase (Maric et al., 2014; Moreno et al., 2014). In conclusion, DNA replication requires CDC48/p97 activity to terminate DNA synthesis by unloading of active CMG complexes.

Comparing CDC48/p97-dependent regulation of replication licensing with termination of replication leaves open questions to be addressed. Moreno et al. (2014) used an experimental system in *Xenopus* egg extracts that affects polyubiquitylation in progressing S phase, thus allowing exclusive analysis of replication termination. In *S. cerevisiae*, the licensing factor Cdt1 is not regulated via proteolysis, but nuclear export, pointing at distinct regulatory mechanisms between unicellular fungi and metazoans (Tanaka and Diﬄey, 2002; Feng and Kipreos, 2003; Kim and Kipreos, 2007). What are the respective cofactors that define CDC48/p97 specificity toward replication termination? In yeast, the ligase complex SCF^Dia2^ catalyzes the ubiquitylation of MCM7 and thus provides the signal for CMG disassembly (Maric et al., 2014; Maculins et al., 2015). The substrate recognition factor Dia2, however, is not obviously conserved outside of the fungi kingdom. In *Xenopus*, release of CMG complex has been linked to the E3 ligase BRCA1, however, is supposed to be specific to stalled replisomes but not termination (Long et al., 2014; Dewar et al., 2015). As such, CDC48/p97-dependent CMG release might be considered as a combined phenotype: aberrant licensing causing stalled forks, in turn requiring active CMG unloading. In *C. elegans* and *Xenopus* egg extracts, CDC48/p97 is linked to the release of CDC-45 and GINS after S phase is completed (Franz et al., 2011, 2016). Herein, CDC48/p97 cooperates with the cofactors UFD-1-NPL-4 and UBXN-3/FAF1, however, neither the depletion of *ufd-1, npl-4*, nor *ubxn-3* resulted in persistent chromatin-association of MCM subunits (Franz et al., 2016). These observations support the idea that CMG disassembly is more complex involving dedicated CDC48/p97 cofactors separately targeting MCM or CDC45-GINS. It has been speculated that a ring-shaped GINS molecule embraces DNA (Kubota et al., 2003; Boskovic et al., 2007), thus chromatin release might be regulated independently from MCM. Alternatively, CMG disassembly might be differentially regulated in invertebrates and vertebrates. In human cells, unloading of MCM complexes has been linked to the deubiquitylating activity of USP7 (Jagannathan et al., 2014), implicating that regulated MCM release might involve editing of ubiquitin chains. Whether USP7 activity is indeed coordinated by CDC48/p97 remains to be elucidated.

## Protein Dynamics at Sites of DNA Repair

DNA damage poses a major threat to cells. Besides defects in replication, additional intrinsic incidents may threaten chromatin integrity. Hence damage may originate from hydrolytic reactions or reactive oxygen species (ROS). However, also extrinsic and highly carcinogenic sources like UV exposure or tobacco products harm DNA. If unrepaired, DNA damage leads to accumulation of mutations or chromosomal aberrations and promotes genome instability, the cause for many diseases including cancer (Jackson and Bartek, 2009; Ciccia and Elledge, 2010). Specific to the damaging agent, the recognition of the damage and the molecular mechanism for its removal are diverse. One unifying feature of all repair pathways, however, is the progression through specific phases of damage recognition, effective repair, and finally resolution of repair intermediates. The underlying DNA damage response (DDR) triggers the dynamic and hierarchically ordered assembly and disassembly of repair factors on the chromatin (Hoeijmakers, 2001). CDC48/p97 plays a central role in various DNA repair scenarios and specialized cofactors provide mechanistic regulatory insight (**Figure 1**; **Table 1**). Please also see latest review articles on this topic (Vaz et al., 2013; Dantuma et al., 2014; Brinkmann et al., 2015; Dantuma and van Attikum, 2016).

### CDC48/p97 Activity in Processing of DNA Double Strand Breaks

The most detrimental DNA lesions are double strand breaks (DSBs) since inadequate fusion of loose ends can give rise to considerable chromosome rearrangements, duplications, or deletions and hence are a severe threat for genome integrity (Hoeijmakers, 2009). Two main pathways known to repair DSBs operate differently. One is comparably simple by ligating the loose ends back together in a reaction called non-homologous end-joining (NHEJ). The other, termed homologous recombination (HR) is a much more concise pathway, using a homologous template for reestablishing the undamaged state (Hoeijmakers, 2001; San Filippo et al., 2008; Lieber, 2010; Mehta and Haber, 2014; Kowalczykowski, 2015).

CDC48/p97 was first implicated in DNA repair by finding that it gets phosphorylated at S^784^ upon DNA damage induction (Livingstone et al., 2005). Indeed DNA-dependent protein kinase, catalytic subunit (PKcs), one of the kinases mediating this PTM (Livingstone et al., 2005), directly interacts with CDC48/p97 upon ubiquitylation (Jiang et al., 2013). DNA-PKcs associates with a heterodimer of Ku70-Ku80 at DSBs and initiates NHEJ (Wang et al., 1994; Davis et al., 2014). CDC48/p97 acts here to restrict DNA PKcs occupancy on DNA by handing it over to proteasomal turnover. In this glioma cell model, loss of CDC48/p97 improves repair efficiency temporally (Jiang et al., 2013). Conversely, other studies described an increase in sensitivity toward DNA damage and subsequent genome instability when CDC48/p97 activity is limited (Acs et al., 2011; Meerang et al., 2011; Raman et al., 2011). Upon DNA DSB induction a well-studied signaling cascade is commenced, involving initial phosphorylation steps but subsequent engagement of the ubiquitin and SUMO machinery to establish binding sites for specific signaling proteins like BRCA1 and 53BP1 (Bekker-Jensen and Mailand, 2011; Polo and Jackson, 2011). This process requires tight regulation; however, ubiquitylation does not only serve as a binding platform orchestrating the recruitment of specific interaction partners. Instead also poly-(K48)-linked ubiquitin chains, which trigger proteasomal degradation (Dammer et al., 2011), were identified at DDR sites that strongly accumulate in CDC48/p97 depleted cells (Meerang et al., 2011). This observation indicates the requirement for CDC48/p97 to remove K48-ubiquitylated proteins from break sites and possibly allow recruitment of downstream factors. In fact, loss of CDC48/p97 function seems to have broad impact on recruitment of repair proteins. After treating cells with ionizing irradiation, CDC48/p97 depletion attenuates recruitment of 53BP1, BRCA1, and abolishes loading of RAD51 to repair sites (Meerang et al., 2011). Mechanistically it remained unclear how CDC48/p97 promotes recruitment of downstream proteins. A recent study highlighted that CDC48/p97 specifically enables recruitment of 53BP1 to DSBs induced by micro-irradiation. 53BP1 association with DSBs is dependent on the ubiquitin cascade but does itself not bind to ubiquitin (Botuyan et al., 2006). Here yet again a switch in signaling molecules has to be implemented. 53BP1 binds to H4K20me2, a histone mark that is initially occupied by L3MBTL1 (Min et al., 2007). Upon ubiquitylation, L3MBTL1 is primed for extraction by CDC48/p97 together with its cofactors NPL4 and UFD1. Only then 53BP1 is able to bind its designated recruitment site and the repair process can be pursued (Acs et al., 2011). Even though different outcomes of CDC48/p97 activity at damage loci are described, both emphasize the requirement of CDC48/p97 to extract ubiquitylated target proteins from DNA damage signaling sites to facilitate further repair steps.

### CDC48/p97 Functions as SUMO-dependent Segregase to Provide Genome Stability

Aside ubiquitin-conjugates CDC48/p97 and its cofactor UFD1 both recognize SUMOylated target proteins directly (Nie et al., 2012). A functional relevance of CDC48/p97 exclusively targeting SUMO-conjugates was first described in the assembly of downstream effector proteins during DNA repair (Bergink et al., 2013). During HR the essential recombinase RAD51 forms long filaments on the two single stranded loose DNA ends, which enable scanning and approaching the homologous sequence (Holthausen et al., 2010; Forget and Kowalczykowski, 2012). Its DNA association needs to be tightly controlled, since hyper-recombination is highly cytotoxic; at the same time RAD51 is essential for recombination (Tsuzuki et al., 1996; Sonoda et al., 1998; Richardson et al., 2004; Klein, 2008). In yeast, SUMO-conjugated Rad52 interacts with and aides Rad51 loading onto DNA when engaged in HR. Interestingly, CDC48/p97^Ufd1^ has direct binding affinity toward the same SUMOylated lysine on Rad52 hence counterbalancing recombination events mediated by Rad51 (Bergink et al., 2013). This finding highlights a function of CDC48/p97 independent of ubiquitin and implies that competitive binding to SUMO can promote segregation activity.

In addition to minimizing Rad51-Rad52 interaction, CDC48/p97 plays a more global role in the regulation of SUMO-conjugates at the chromatin (Køhler et al., 2015). SUMOylation was established as another layer of regulation at DSB sites that enables thorough repair (Hardeland et al., 2002; Cremona et al., 2012; Jackson and Durocher, 2013). Mutations in SUMO related proteins lead to genomic instability, but again, inappropriate retention of SUMOylated proteins on DNA also impedes accurate repair since recruitment of downstream factors is strongly reduced (Psakhye and Jentsch, 2012). Interestingly, the CDC48/p97^Ufd1-Npl4^ complex has been implicated in this specific extraction as well. Ufd1 harbors a SUMO interaction motif (SIM) by which it directly binds SUMO (Nie et al., 2012). Additionally, specific ubiquitin ligases target SUMOylated proteins for ubiquitylation (SUMO-targeted ubiquitin ligases: STUbLs). CDC48/p97 together with its cofactors is thus recruited via a dual mechanism consisting of ubiquitin and SUMO, thereby facilitating extraction and possibly degradation of SUMOylated proteins at DNA damage sites (Nie et al., 2012). UFD1 takes an important role as cofactor of CDC48/p97. In addition to its direct binding to SUMO, a physical as well as functional interaction of Ufd1 with the STUbL Rfp1 (RNF4 ortholog) or the SUMO E3 ligase Pli1 (PIAS1) was shown. The concerted action of these proteins leads to ordered removal of SUMOylated proteins at damage site, again their inappropriate retention by loss of one of the factors entails genomic instability (Køhler et al., 2013). This example nicely highlights CDC48/p97s function as a molecular switch. It provides a platform for a variety of functionally distinct proteins that together lead to precisely coordinated CDC48/p97-dependent chromatin extraction of client proteins (**Figure 1**; **Table 1**).

An analogous mechanism was described for DNA repair by the Fanconi anemia pathway. After replication block, Fanconi anemia pathway becomes active to promote fork restart by initiating translesion synthesis and damage removal (Haynes et al., 2015). Two central components, FANCI and FANCD, are SUMOylated upon fork stalling. As described for other SUMOylated proteins, they are targeted for degradation by RNF4 mediated ubiquitylation and subsequent mobilization from DNA by the CDC48/p97 complex (Gibbs-Seymour et al., 2015). Here CDC48/p97 acts jointly with DVC1. Degradation of FANCI and FANCD is impaired in RNF4 mutants, highlighting that in this case ubiquitin binding of the CDC48/p97^DV C1^ complex is necessary.

### Processing of DNA-protein Crosslinks

Proteins that are crosslinked to DNA or chromatin are a specialized form of chromatin modification, which can arise from metabolic sources or external insults such as reactive aldehydes, UV-light, or catalytic intermediates, e.g., upon Topoisomerase 1 inhibition (Duxin and Walter, 2015; Stingele and Jentsch, 2015; Stingele et al., 2015). DPCs result in stalling of RNA and DNA polymerases and thus impact on a variety of cellular processes. Consequently, DPCs need to be removed in a regulated manner that involves incomplete proteolytic digestion. Subsequently, the processed DPC remnant can be bypassed by a specialized translesion polymerase (Duxin et al., 2014), whereas the DPC remnant itself is considered to be eventually removed by base excision repair. Intriguingly, a DVC1-related protease acts as CDC48/p97 cofactor and harbors protease activity to catalyze DPC processing in yeast (Stingele et al., 2014, 2015; **Figure 1**; **Table 1**). Wss1 protease activity becomes particularly activated upon DNA binding, where it digests DPCs including covalently bound Topoisomerase 1, other chromatin-bound proteins as well as itself for inactivation (Stingele et al., 2014; Balakirev et al., 2015). Interestingly, Wss1 specifically targets SUMO-conjugates on chromatin via its SUMO-interaction motif (Mullen et al., 2010; Stingele et al., 2014; Balakirev et al., 2015). In contrast DVC1 is directed toward ubiquitin-conjugates and is linked to the PCNA sliding clamp (Centore et al., 2012; Davis et al., 2012; Juhasz et al., 2012; Mosbech et al., 2012; Gibbs-Seymour et al., 2015). In case of replication fork stalling-induced extraction of the Fanconi anemia ID complex, the SUMO-dependent ubiquitin E3 ligase RNF4 is central for the underlying signaling (Gibbs-Seymour et al., 2015), supporting the idea that DVC1 regulation in metazoans is multilayered involving both SUMO and ubiquitin. Although a chromatin-directed protease activity of DVC1 awaits to be verified, it is feasible that DVC1 and Wss1 represent functional equivalents. This might explain initial observations that DVC1 is required for the removal of translesion polymerase after UV-lesion bypass (Centore et al., 2012; Davis et al., 2012; Ghosal et al., 2012; Juhasz et al., 2012; Mosbech et al., 2012; Kim et al., 2013), which is probably linked to attenuated processing of DPCs (Duxin et al., 2014; Duxin and Walter, 2015). Recently, the CDC48 cofactor Doa1 (also known as UFD3 or PLAP) was shown to be present in CDC48/p97^Wss1^ complexes (Balakirev et al., 2015). In this study, genotoxic stress resulted in nuclear GFP-Wss1 punctae, consistent with the formation of DNA repair foci. This is in line with formation of nuclear DVC1 foci upon exposure to genotoxic stress by HU, UV-light, or laser microirradiation in *C. elegans* and mammalian cells (Davis et al., 2012; Mosbech et al., 2012). Moreover, GFP-Wss1 translocates to the vacuole upon damage induction, pointing at a putative role of lysosomal degradation in Wss1-mediated DDR involving the Doa1 cofactor.

To date, the mechanistic role of CDC48/p97 in Wss1-dependent DDR is still obscure. Upon genotoxic insults DVC1/Wss1 recruits CDC48/p97 to the damaged site, pointing at an adaptor-like function promoting segregase activity (Centore et al., 2012; Davis et al., 2012; Mosbech et al., 2012; Gibbs-Seymour et al., 2015). It appears likely, that DVC1/Wss1’s intrinsic protease activity is particularly important in cases of DPCs, when CDC48/p97 segregase is not capable of processing the substrate due to covalent linkage to the DNA (Stingele et al., 2014). The observation that Wss1 harbors SUMO-ligase (Balakirev et al., 2015) as well as isopeptidase activity (Mullen et al., 2010) suggests that Wss1 function might include additional layers of regulation.

### CDC48/p97 Dependent Extraction in UV Induced DNA Damage Repair

Not only replication block but also obstruction of transcription poses a major threat to DNA. Frequent sources of transcription fork stalling are bulky UV lesions (Mullenders, 2015). To avoid stalling of forks, the cell probes constantly for these helix distortions via the global genome nucleotide excision repair (GG-NER) pathway that is active on both DNA templates. Here, XPC together with UV-DDB complex (UV-DNA damage binding protein) consisting of DDB1 and DDB2 detect lesions and initiate repair (Hoogstraten et al., 2008; Marteijn et al., 2014). To proceed with the excision reaction, these factors need to be removed from chromatin. CDC48/p97 in complex with NPL4-UFD1 and UBXD7 regulates the chromatin association of XPC and DDB2. Ubiquitin dependent extraction by CDC48/p97 allows their proteasomal degradation. Depletion of CDC48/p97 promotes retention of those factors and ultimately leads to genomic aberrations (Puumalainen et al., 2014). In contrast, the DUB USP7 was identified to counteract CDC48/p97 dependent extraction of XPC. It shortens the ubiquitin chain on the target protein, thereby removing the signal for extraction and possible degradation (He et al., 2014).

In case RNA Polymerase II (RNA Pol II) encounters such lesions on the actively transcribed strand, transcription coupled NER (TC-NER) is initiated (Spivak and Ganesan, 2014). During TC-NER CSB is involved in repair initiation (Fousteri et al., 2006; Anindya et al., 2010). Similar to XPC and DDB2 degradation in GG-NER, CDC48/p97-dependent proteolysis of CSB is required to facilitate progression of DNA repair upon UV-irradiation (He et al., 2016). To this end, CSB removal from chromatin is mediated by the cofactors UFD1 and UBXN7 (He et al., 2016). Both sub-pathways, GG-NER and TC-NER have different initiation signals, but merge into one mutual pathway after initial processing. When the injured DNA is excised the gap of 22–30 nucleotides needs to be sealed again. This is accomplished by replication proteins, including PCNA, a DNA polymerase, and subsequent ligation (Marteijn et al., 2014; Mullenders, 2015). In this context, CDC48/p97 is associated with removal and ensuing proteasomal degradation of CDT1 and histone methyl transferase SET8 (Raman et al., 2011). Chromatin association of the two proteins is tightly regulated not only during replication but also upon repair to avoid unscheduled replication initiation (Senga et al., 2006). Binding to the PCNA interaction protein motif degron (PIP degron) of PCNA generally primes target proteins for ubiquitylation by CRL4^Cdt2^ (Havens and Walter, 2009). Hence, after UV damage, CDC48/p97 extracts ubiquitylated CDT1 and SET8 from damaged chromatin and sends both substrates for destruction by the proteasome (Raman et al., 2011).

Normally the cell favors preserving RNA Pol II upon stalling; this is achieved by RNA Pol II backtracking, which allows repair proteins to access the lesion (Epshtein et al., 2014). As a last resort, when NER cannot be executed, RNA Pol II is removed and degraded to prevent even more severe damage (Wilson et al., 2013). Upon UV irradiation, degradation of the largest subunit of RNA Pol II Rpb1 is facilitated by ubiquitin dependent extraction. Herein, CDC48/p97 cooperates with its cofactors Ufd1 and Npl4, as well as with Ubx5 (UBXN7; Verma et al., 2011), a cofactor that is also associated with NER dependent protein extraction in humans.

## Additional Function of CDC48/p97 in Chromosome Biology

In contrast to the load of DNA damage another determinant of cellular aging is the shortening of chromosome ends, the telomeres, with consecutive cell divisions. Interestingly, a function of CDC48/p97 in the regulation of telomere length has recently been proposed based on the identification of Cdc13 and Est1 as substrate proteins (Baek et al., 2012; Lin et al., 2015; **Figure 1**; **Table 1**). Both, Cdc13 and Est1 are key regulators of telomere replication in yeast. Baek et al. (2012) showed that CDC48/p97 cooperates with the Vms1 (ANKZF1) cofactor in the proteolytic turnover of Cdc13. Interestingly, Cdc13 destruction appears to involve both proteolytic routes, the proteasome and the lysosome. In contrast Lin et al. (2015) propose that CDC48/p97 together with Ufd1-Npl4 and the ubiquitin E3 ligase Ufd4 (TRIP12) cooperate to adjust telomere length by limiting the abundance of mono-ubiquitylated Est1. Consequently, CDC48/p97 inactivation results in shortened telomeres, presumably due to inefficient telomerase upon Cdc13 and Est1 accumulation.

In addition to DNA synthesis and repair pathways, CDC48/p97 plays pivotal roles during sister-chromatid segregation. Faulty segregation of chromatids during mitosis is a significant source of copy number variations, large chromosomal aberrations, or chromosome destruction. In fact, a key regulator of mitosis, the kinase Aurora-B, was the first chromatin-associated substrate of CDC48/p97 to be identified (Ramadan et al., 2007; **Figure 1**; **Table 1**). Interestingly, CDC48/p97^Ufd1-Npl4^ restricts Aurora-B activity to promote chromosome congression or chromatin relaxation at distinct time-points during mitosis, as well as chromosome segregation during meiotic division (Ramadan et al., 2007; Dobrynin et al., 2011; Sasagawa et al., 2012). The involvement of cofactors in the regulation of Aurora-B during meiosis I, however, remains to be defined. In yeast cells, CDC48/p97 and its cofactor Ubx4 are involved in the nuclear distribution of proteasomes, thus defining protein degradation during anaphase (Chien and Chen, 2013), implicating that additional CDC48/p97 substrates await to be identified in the context of mitosis.

Aside DNA metabolism CDC48/p97 executes critical function in the regulation of DNA-dependent RNA synthesis. Of note, failure in accurate regulation of transcription factors controlling metabolism and cell proliferation are associated with oncogenesis (Maneix and Catic, 2016). Chromatin-dependent activity of CDC48/p97 in the regulation of gene expression is especially interesting, as it does not involve subsequent protein degradation (Wilcox and Laney, 2009; Ndoja et al., 2014; **Figure 1**; **Table 1**). In yeast, CDC48/p97 controls rapid switch in gene expression through non-proteolytic release of transcription factors from the chromatin. Intriguingly, individual transcription factors that are regulated by CDC48/p97 represent transcriptional activators as well as repressors (Wilcox and Laney, 2009; Ndoja et al., 2014). Thus, CDC48/p97 is capable of initiating fast response toward transcriptional stimuli through attenuation or activation of gene expression. In *S. cerevisiae* the Ufd1-Npl4 cofactor and Doa10 are linked to transcriptional regulation by CDC48/p97 (Wilcox and Laney, 2009; Ndoja et al., 2014). In mammalian cells CDC48/p97 together with the cofactor UBXN7 and the CUL2-VHL ubiquitin ligase mediates the proteolytic inactivation of HIF1α transcription factor, suggesting a critical role of CDC48/p97 in the cellular response toward hypoxia (Alexandru et al., 2008). It remains to be shown, whether CDC48/p97-mediated regulation of HIF1α occurs on chromatin. In contrast to the extraction of transcription factors, CDC48/p97 is also implicated in chromatin remodeling. In yeast CDC48 together with Ubx3 (UBXD8) is required for the mono-ubiquitylation on histone 2B, thus controlling chromatin compaction and presumably differentiation in vertebrates (Bonizec et al., 2014). An alternative pathway controlling gene expression was described in *Arabidopsis*. Here, CDC48/p97^NPL4^ promotes chromatin de-condensation through regulated disassembly of centromeric heterochromatin, resulting in the release of rRNAs, which facilitates ribosome biogenesis (Merai et al., 2014). Here, SUMOylated centromere components including the centromeric histone variant CenH3 trigger chromatin relaxation.

## Concluding Remarks

The described molecular mechanisms illustrate the central role of CDC48/p97 in the dynamic control of protein composition in the chromatin environment (**Figure 1**; **Table 1**). CDC48/p97 operates at the intersection of two major signaling pathways at the chromatin, ubiquitylation and SUMOylation. It is currently unclear whether both signaling pathways initiate separate mechanisms (Meerang et al., 2011; Bergink et al., 2013) or whether both pathways eventually converge into a common pathway (Nie et al., 2012; Køhler et al., 2013; Gibbs-Seymour et al., 2015) with CDC48/p97 as nodal point (**Figure 1**). It is feasible, however, that independent and shared signaling pathways act in parallel. Whereas ubiquitin- and SUMO-signaling are essential in mediating timely response toward genotoxic insults, both modifications need to be removed eventually to restore genome integrity. Regarding ubiquitin signaling, CDC48/p97-dependent processing of substrates on chromatin has exclusively been shown to target either K48-linked ubiquitin chains (Ramadan et al., 2007; Meerang et al., 2011; Maric et al., 2014; Moreno et al., 2014) or mono-ubiquitin (Lin et al., 2015). Whether other linkage-types are involved in CDC48/p97 regulation at the chromatin remains to be deciphered. Mono-ubiquitin and K63-linked chains are essential in the initial signaling of the molecular response to DSBs (Dantuma and van Attikum, 2016). Thus, the recognition of these modifications by CDC48/p97 may provide additional mechanistic insights in early events at DSBs. In this context, it will be of interest to address which cofactors are involved in either linkage-specific recognition or editing of linkage-types (**Figure 1**). Due to its diverse functions, global CDC48/p97 inhibition causes pleiotropic defects on chromosome biology. Thus it will be crucial to identify the cofactors that direct specificity and discriminate between distinct pathways. Although CDC48/p97 inhibitors are tested in clinical trial studies with promising properties (Anderson et al., 2015), a more specific targeting might be applicable through the selective manipulation of cofactors. The identification of substrate proteins targeted by CDC48/p97 will allow future studies to uncover the underlying molecular mechanisms in more detail, pointing out commons and differences.

## Author Contributions

AF and TH elaborated the concept of the manuscript. AF, LA, and TH wrote the manuscript. All authors discussed the text and commented on the manuscript.

## Conflict of Interest Statement

The authors declare that the research was conducted in the absence of any commercial or financial relationships that could be construed as a potential conflict of interest.
